# Molecular Classification Models for Triple Negative Breast Cancer Subtype Using Machine Learning

**DOI:** 10.3390/jpm11090881

**Published:** 2021-09-01

**Authors:** Rassanee Bissanum, Sitthichok Chaichulee, Rawikant Kamolphiwong, Raphatphorn Navakanitworakul, Kanyanatt Kanokwiroon

**Affiliations:** Department of Biomedical Sciences and Biomedical Engineering, Faculty of Medicine, Prince of Songkla University, Hat Yai, Songkhla 90110, Thailand; rassanee.b@gmail.com (R.B.); sitthichok.c@psu.ac.th (S.C.); k.rawikant@gmail.com (R.K.)

**Keywords:** TNBC subtype, machine learning, microarray, gene expression profile

## Abstract

Triple negative breast cancer (TNBC) lacks well-defined molecular targets and is highly heterogenous, making treatment challenging. Using gene expression analysis, TNBC has been classified into four different subtypes: basal-like immune-activated (BLIA), basal-like immune-suppressed (BLIS), mesenchymal (MES), and luminal androgen receptor (LAR). However, there is currently no standardized method for classifying TNBC subtypes. We attempted to define a gene signature for each subtype, and to develop a classification method based on machine learning (ML) for TNBC subtyping. In these experiments, gene expression microarray data for TNBC patients were downloaded from the Gene Expression Omnibus database. Differentially expressed genes unique to 198 known TNBC cases were identified and selected as a training gene set to train in seven different classification models. We produced a training set consisting of 719 DEGs selected from uniquely expressed genes of all four subtypes. The highest average accuracy of classification of the BLIA, BLIS, MES, and LAR subtypes was achieved by the SVM algorithm (accuracy 95–98.8%; AUC 0.99–1.00). For model validation, we used 334 samples of unknown TNBC subtypes, of which 97 (29.04%), 73 (21.86%), 39 (11.68%) and 59 (17.66%) were predicted to be BLIA, BLIS, MES, and LAR, respectively. However, 66 TNBC samples (19.76%) could not be assigned to any subtype. These samples contained only three upregulated genes (*EN1*, *PROM1*, and *CCL2*). Each TNBC subtype had a unique gene expression pattern, which was confirmed by identification of DEGs and pathway analysis. These results indicated that our training gene set was suitable for development of classification models, and that the SVM algorithm could classify TNBC into four unique subtypes. Accurate and consistent classification of the TNBC subtypes is essential for personalized treatment and prognosis of TNBC.

## 1. Introduction

Triple negative breast cancer (TNBC) is a subset of breast cancer which lacks the expression of estrogen receptor (ER), progesterone receptor (PR), and human epidermal growth factor receptor-2 (HER2). TNBC accounts for 10–20% of breast cancers, and primarily affects women under 40 years old [1]. Due to the heterogeneity of the disease, and the absence of molecular targets, TNBC patients are not sensitive to endocrine or HER2 targeted therapy. Chemotherapy remains a standard treatment for patients with TNBC. This cancer has a poor prognosis and a high rate of relapse and metastasis leading to tumor recurrence [2,3]. Hence, it is important to explore therapeutic targets to improve the outcomes of TNBC. Advances in gene expression microarray analysis have facilitated comprehensive molecular profiling, which can be used to classify TNBC into distinct subtypes [4,5,6,7,8]. According to gene expression signatures, Burstein et al. classified TNBC into four subtypes: basal-like immune-activated (BLIA), basal-like immune-suppressed (BLIS), mesenchymal (MES), and luminal androgen receptor (LAR) [8]. Previous studies have found that each TNBC subtype has different characteristics and responses to neoadjuvant chemotherapy [9,10]. Thus, TNBC subtyping is of value for prioritizing patients for personalized medicine. However, a laboratory tool for classification of TNBC subtype is still under investigation, and has not yet been implemented in the clinic.

Over the past decade, research groups studied the gene signatures of TNBC subtypes using different techniques. Advancement in gene microarray technology have produced datasets with a very large number of genes (features), but a small number of samples. This high dimensionality is a major challenges to the development of classification methods [11,12]. To address these challenges, machine learning (ML) approaches have been used. ML is the study of computer algorithms which improve automatically through experience. It learns from previous data to create the classification, prediction or identify processes [13]. To date, ML-based cancer classification models have been used to predict death outcomes [14], seek new drug mechanisms [15] and identify genes to differentiate TNBC from non-TNBC [16]. However, no reported studies have proposed ML-based classification schemes for classifying TNBC subtypes using gene expression data. The aim of this study was to investigate the potential application of ML to the classification of TNBC subtypes using microarray data derived from the public Gene Expression Omnibus (GEO) database [17]. In the present study, we analyzed a dataset consisting of 198 TNBC patients, to identify a set of upregulated differentially expressed genes (DEGs) among TNBC subtypes. We subsequently used this gene set to develop classification models using seven different ML algorithms: Support Vector Machines (SVM), K-nearest neighbor (KNN), Naïve Bayes (NB), Decision Tree (DT), Ensemble, Linear Discriminant, and Logistic Regression. 

## 2. Results

### 2.1. Identification of DEGs and Feature Selection

**Figure 1:**
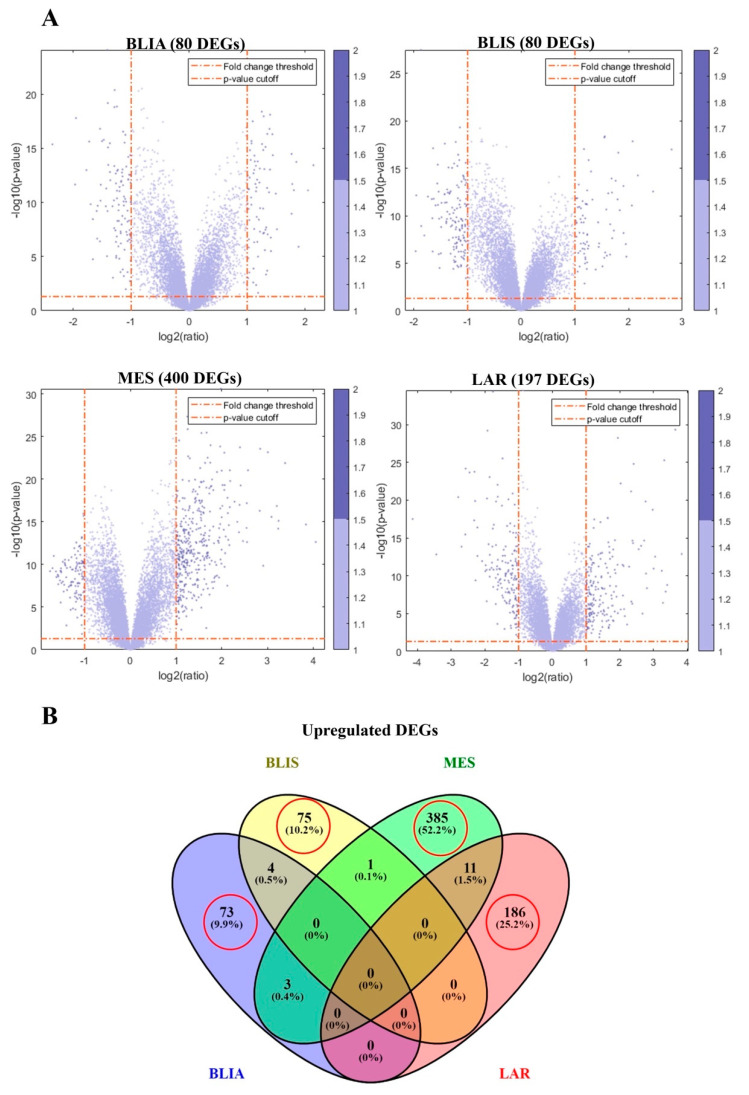
(**A**) Volcano plots show differentially expressed genes (DEGs) among the four triple negaTable 0. and the vertical red dashed lines correspond to log 2-fold change (FC) value. The cut off at *p* < 0.05 and FC > 2 was considered to indicate significantly upregulated DEGs. (**B**) Venn diagram illustrates overlapping upregulated DEGs among the TNBC subtypes. Red circles indicate unique genes found in each TNBC subtype.

In this study, 198 known TNBC cases used as the training set were classified into four TNBC subtypes: BLIA, BLIS, MES, and LAR, containing 54, 60, 47, and 37 cases, respectively. The samples contained the expression profiles of 20,186 genes, making the dataset very high dimensional. Using a large number of genes to train the ML model takes a long time, and may reduce the efficiency of ML. Thus, to train the model and identify the best classifiers, we extracted the genes with upregulated expressed in each TNBC subtype according to the *p*-value and log_2_ fold change cut-offs. We identified 80, 80, 400, and 197 upregulated DEGs in BLIA, BLIS, MES, and LAR, respectively (Figure 1A). The top 20 upregulated DEGs of each subtype are shown in Table 1. We also used Venn diagrams to check the overlap between upregulated DEGs of each subtype, and found 73, 75, 385, and 186 genes which were expressed only in BLIA, BLIS, MES, and LAR, respectively (Figure 1B) (Supplementary Table S1). There were few overlapping upregulated DEGs among the four subtypes. Hence, the 719 DEGs which were only expressed in each subtype were selected as the training gene set for training the classification models.

### 2.2. GO Term and KEGG Pathway Enrichment Analysis of Unique Upregulated DEGs in Each TNBC Subtype

**Figure 2:**
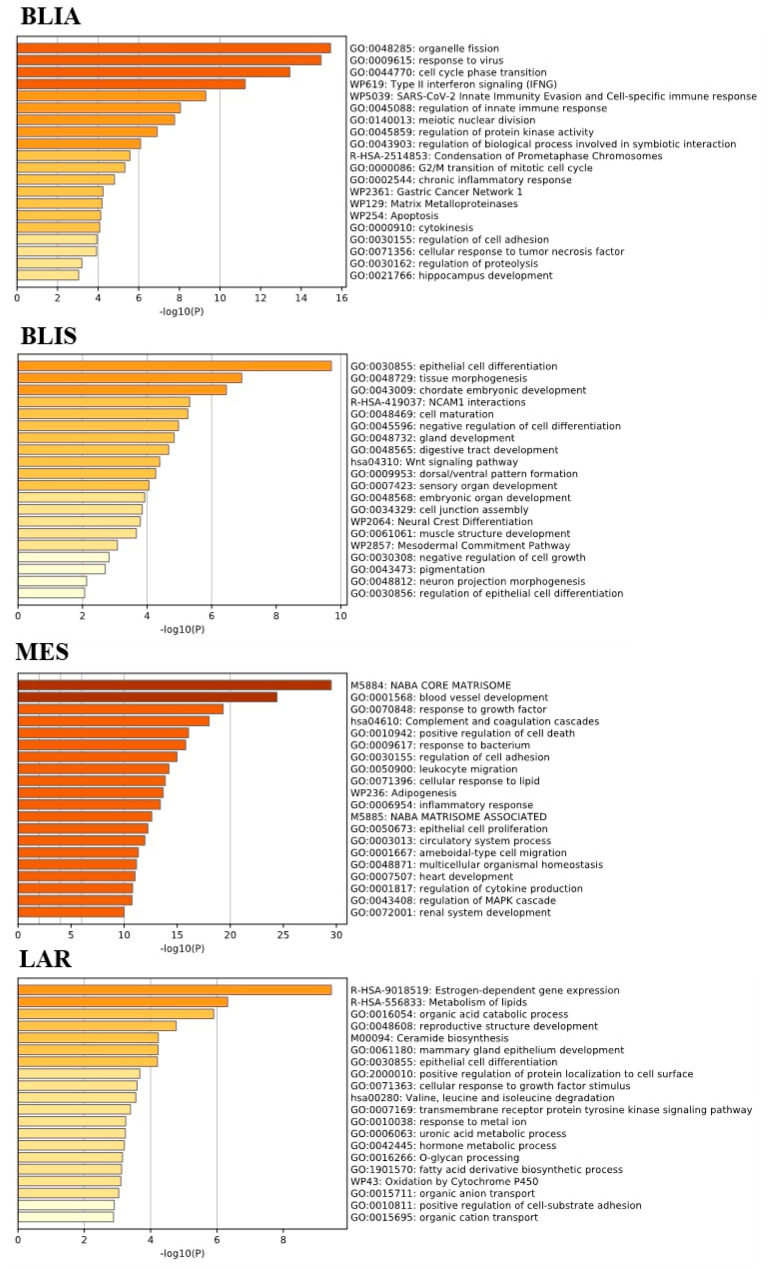
GO term and KEGG pathway enrichment analysis of uniquely upregulated DEGs of each TNBC subtype.

GO function and KEGG pathway enrichment analysis were performed using MetaScape [18] to explore the biological functions of unique upregulated DEGs in each TNBC subtype. DEGs which were only upregulated in the BLIA subtype were significantly enriched in organelle fission, nuclear division, cell cycle phase transition, and immune regulation pathways. These genes were different from those of the BLIS subtype, which were significantly downregulated in immune regulation pathways [5]. In our study, functional enrichment analyses of upregulated DEGs in the BLIS subtype which is one of two basal-like clusters, showed significant enrichment in epithelial cell differentiation, tissue morphogenesis, chordate embryonic development. and the Wnt signaling pathway. In the MES subtype, signal transduction pathways associated with the naba core matrisome, which is an ensemble of genes encoding core extracellular matrix elements including ECM glycoproteins, collagens, and proteoglycans. The upregulated DEGs of the LAR subtype were mainly significantly enriched in estrogen-dependent gene expression, metabolism of lipids, and organic acid catabolic processes. Thus, our results indicated that each TNBC had a unique pattern of gene expression and signaling pathways (Figure 2) (Supplementary Table S2).

### 2.3. Modeling Prediction and Performance Evaluation

In this section, all of the unique upregulated DEGs of each TNBC subtype were selected as the training set for classification and prediction using the MATLAB 2020a environment [19]. To choose the best model, we trained a selection of models, including SVM, KNN, NB, DT, Ensemble, Linear Discriminant, and Logistic Regression. We trained all of the models using five-fold cross validation, to protect against overfitting. The training data in our experiment were divided into five sets of similar size, and four of them were used in turn as the training set. One set was used as the test set to evaluate the model. In terms of model accuracy, the prediction models ranged from 61.5% for logistic regression, to 98.8% for SVM. The SVM algorithm was the best classification model, with the highest average accuracy of 95.7, 95.6, 95.0, and 98.8% for the BLIA, BLIS, MES, and LAR subtypes, respectively. The experimental results are presented in Table 2 (Supplementary Table S3). 

**Figure 3:**
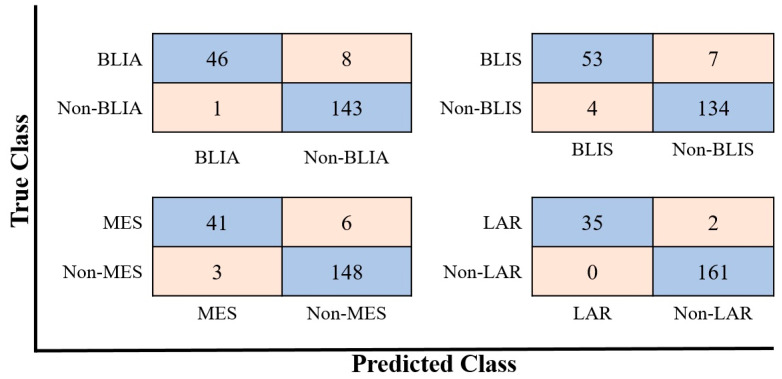
Confusion matrixes of the best performing models using the SVM algorithms.

**Figure 4:**
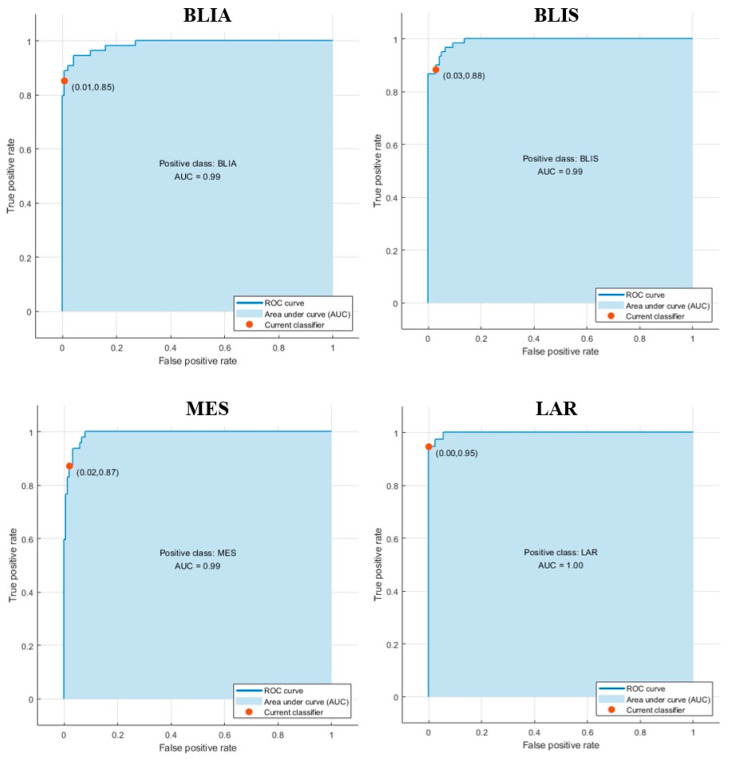
Performance evaluation of the best classification models using ROC curves and AUC values for BLIA, BLIS, MES, and LAR.

Since the number of TNBC patients in each subtype was not balanced, the accuracy may not reflect the performance of the ML algorithms. Therefore, to prevent misleading interpretation of our results, the F1 score, which is the harmonic mean of precision and recall, was also considered. The BLIA, BLIS, MES, and LAR models exhibited F1-scores of 0.91, 0.91, 0.90, and 0.97, respectively. The LAR model was able to predict non-LAR patients correctly, resulting in a recall (sensitivity) of 1.00 (100%) (Figure 3, Table 3). The area under the ROC curve (AUC) value was also used to evaluate the model performance. The SVM model of LAR exhibited the highest AUC, of 1.00, compared to BLIA, BLIS, and MES, with an AUC of 0.99 (Figure 4). Our result showed that a training gene set and ML algorithms could classify TNBC with high accuracy. 

### 2.4. Testing on Independent Cohorts Demonstrated the Generalizability of the Classification Model

**Figure 5:**
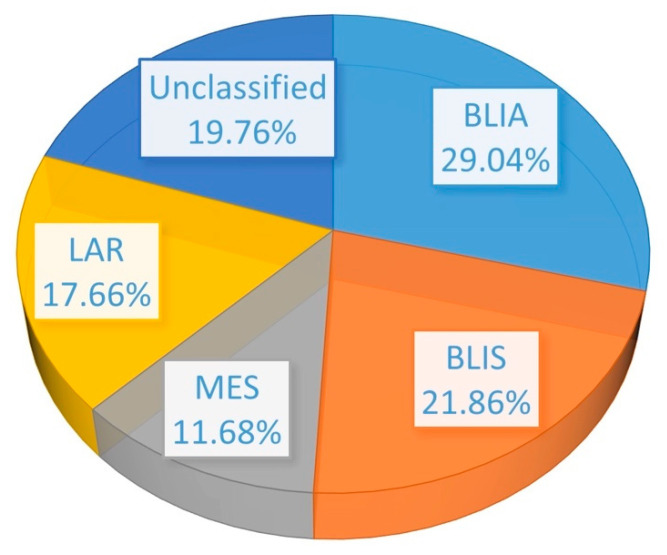
Pie chart showing the percentage of TNBC patients in the test set (334 cases) that were classified into four subtypes or unclassified.

**Figure 6:**
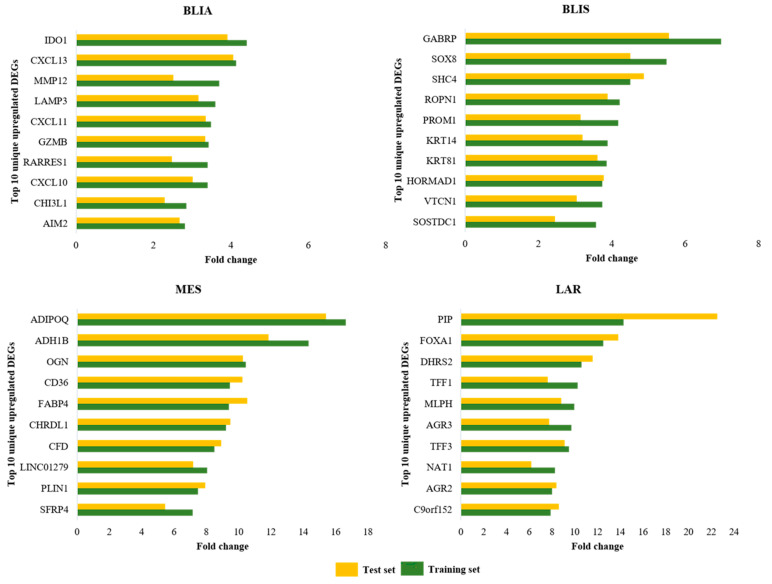
Comparison of fold change of the top 10 upregulated DEGs between the test and training sets of the BLIA, BLIS, MES, and LAR subtypes.

The model was then evaluated on independent datasets of TNBC subtypes that had never been used in the training process. In model validation of 334 unknown TNBC samples, 97 (29.04%), 73 (21.86%), 39 (11.68%) and 59 (17.66%) were predicted to be BLIA, BLIS, MES, and LAR, respectively. In addition, 66 TNBC samples (19.76%) could not be predicted as any subtype, and were defined as unclassified samples (Figure 5) **(**Supplementary Table S4). Then, the upregulated DEGs of the test set were compared with those of the training set, to confirm the accuracy of each model. Our result showed that the DEG comparisons of the individual subtypes were quite similar between the training and the test set (Figure 6) (Supplementary Table S5). The unclassified samples included only three upregulated genes, including *EN1, PROM1,* and *CCL2* among all TNBC subtypes (Table 4). 

## 3. Discussion

TNBC is a more aggressive and highly heterogeneous disease than other type of breast cancer [20,21]. TNBC patients do not benefit from targeted therapies such as endocrine therapy or trastuzumab, due to the absence of ER, PR, or HER2. TNBC patients have poorer survival and prognosis than other breast cancer types after chemotherapy [22,23]. Due to its high heterogeneity, TNBC can be classified into different subtypes [6,7,8,24]. Burstein et al. divided TNBC into four subtypes, BLIA, BLIS, MES, and LAR [8]. Some studies have found that TNBC subtypes have different prognosis and responses to neoadjuvant chemotherapy. LAR patients achieved the lowest pathologic complete response (pCR), but showed the best overall survival rate and delayed recurrence when compared with the other subtypes [9]. Patients with Basal-like 1 subtype (Lehmann subtyping) exhibited the highest pCR to carboplatin containing regimens [25]. TNBC subtyping can be used as a predictor of pCR, and may impact decision pertaining to treatment of TNBC. Thus, classification tools are needed to classify TNBC subtypes. However, the classification of TNBC subtypes has not been routinely used in clinical practice. The main goal of our study was to use public gene expression data to develop a tool for TNBC subtyping, using ML. 

In this study, microarray gene expression data was downloaded from the GEO database. To enhance the efficacy of ML, gene signatures were selected from genes expressed only in each subtype, as identified using a cutoff of FC > 2 and *p*-value < 0.05. There were a few overlapping DEGs among subtypes, because they shared some similar characteristics. For example, BLIA and BLIS displayed basal-like characteristics. The training set 719 DEGs was used to train the prediction models. The highest average accuracy of a classifier for BLIA, BLIS, MES, and LAR subtype was the SVM algorithm (accuracy 95–98.8%; AUC 0.99–1.00). The performance was evaluated based on five-fold cross validation. SVM is a supervised ML algorithm based on the idea of maximizing the margins between different classes. Our results were consistent with those of previous studies. Asri et al. found that SVM showed the highest accuracy (97.13%) and lowest error rate in the classification of breast cancer, using the Wisconsin Breast Cancer datasets [26]. Wu et al. also found that the SVM algorithm could accurately classify breast cancer into TNBC and non-TNBC, and had fewer misclassification errors than the other ML algorithms [27]. Nindrea et al. confirmed that the SVM algorithm produced better accuracy of breast cancer risk calculation than other ML algorithms [28]. 

To ensure accurate subtype prediction, we compared the upregulated DEGs of each subtype between the training and test sets. We observed that predicted TNBC samples displayed upregulated DEGs that corresponded with those in the training set and in other reports [8]. The different TNBC subtypes exhibited different unique gene expression and signaling pathways. These data could be used to guide therapeutic decisions. The BLIA subtype showed high expression of genes related to the immune system, and therefore may be sensitive to immune checkpoint inhibitor treatments for BLIA. For the MES subtype, upregulated DEGs were associated with extracellular structure, extracellular matrix organization, growth factors, and blood vessel development. Therefore, patients with the MES subtype might be susceptible to anti-angiogenic therapy [29]. The LAR subtype showed significantly upregulated DEGs enriched in estrogen-dependent gene expression, including androgen receptors (AR). AR was expressed at a lower rate in other TNBC subtypes. The LAR model had the highest accuracy, 98.8%, and a recall of 1.0. AR could therefore be used as a novel therapeutic target for the LAR subtype. The use of enzalutamide, an androgen receptor inhibitor, is currently being explored in TNBC patients who express the androgen receptor (NCT01889238) [30,31,32].

The unclassified samples, which were not predicted as any subtype, had only three upregulated genes. *EN1, PROM1*, and *CCL2* were found as upregulated DEGs of BLIS, BLIA&BLIS, and MES, respectively. These patients did not express the unique gene pattern; thus the classification model was unable to identify them into any subtypes. These findings indicated that the training gene set could discriminate between TNBC subtypes. However, further study is needed to investigate the unclassified subtypes. The integration of gene expression analysis with genomic, epigenetic, and microRNA data may lead to improvement of the efficacy of ML classification tools. TNBC subtyping identified the unique patterns of gene expression for each subtype, and could be used for guiding therapeutic choices, and also for the development of potential therapeutic targets for TNBC patients. 

Our discovery phase, the training gene set consisted of 719 DEGs which were high number to train the prediction model. Minimize the number of unique genes in training gene set with high accuracy is challenging. Gene signatures selection will be considered on gene (feature) importance score based on how useful they are in classification model. This will be a practical method to select a few gene signatures for further validation in clinical samples using qPCR. It would be beneficial to utilize the RNAseq data with our ML models in the future.

## 4. Materials and Methods 

### 4.1. Data Sources and Preprocessing

**Figure 7:**
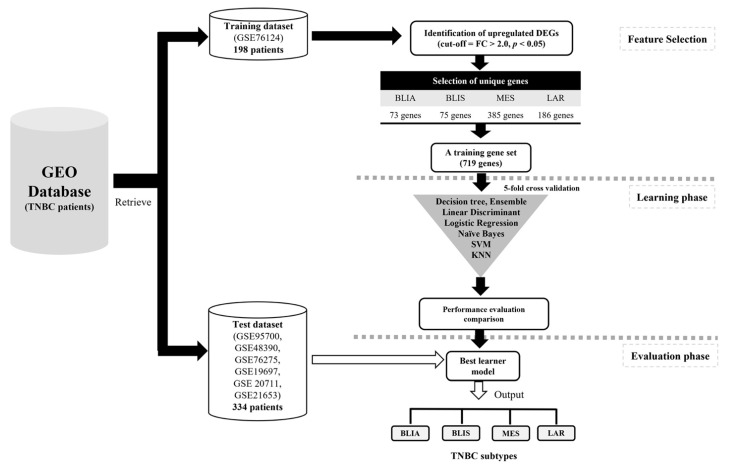
Machine learning analysis workflow for the classification of TNBC subtypes.

The overall design and execution strategy used in this study is presented in Figure 7. We downloaded the seven microarray gene expression profile datasets (GSE76124, GSE95700, GSE48390, GSE76275, GSE19697, GSE 20,711 and GSE21653) from the GEO database (https://www.ncbi.nlm.nih.gov/geo/, accessed on 1 May 2021). Our experimental dataset consisted of 532 TNBC cases, of which 198 TNBC cases were assigned as a training cohort and 334 TNBC cases were assigned as a test cohort. All seven datasets were based on platform GPL570 (Affymetrix Human Genome U133 Plus 2.0 Array) and are freely available online. No additional ethics review was required for the *in silico* analysis of these data sets, because this study did not involve any experiments on humans performed by any of the authors. The raw Affymetrix cell intensity files (.CEL files) of all datasets were processed for normalization, background correction, and log_2_-transformation using robust multi-array average from the R/Bioconductor package affy [33]. 

### 4.2. Identification of DEGs and Feature Selection

The volcano plot which was generated by mavolcanoplot in MATLAB was used to identify DEGs with the most predictive power among TNBC subtypes. In this experiment, known TNBC cases from a previous study [8] were assigned to four TNBC subtypes: BLIA, BLIS, MES, and LAR. These cases were used as training data. We considered *p*-value < 0.05 and a log_2_ (fold change) >2 to indicate statistically significant upregulated DEGs of each TNBC subtype. All upregulated DEGs were then plotted as a Venn diagram, to check for overlapping genes among subtypes. For feature selection, all upregulated DEGs which were expressed in only one of the four subtypes were selected as the gene set for training the prediction models.

### 4.3. Functional and Pathway Enrichment Analysis 

A functional enrichment analysis of the unique upregulated DEGs of each TNBC subtype was performed using the Metascape software (http://metascape.org/, accessed on 1 May 2021) [18]. Functional enrichment was performed using three categories of GO terms: biological process, molecular function and cellular component (CC). In addition, KEGG pathways, Reactome Gene Sets, and CORUM were used as sources of pathway, gene network, and process enrichment analysis [34,35,36]. Terms with a *p*-value of <0.01, a minimum count of 3, and an enrichment factor of >1.5 were collected and grouped into clusters based on their membership similarities. 

### 4.4. Model Construction

Seven different classification models, SVM, KNN, NB, DT, Ensemble, Linear Discriminant, and Logistic Regression, were used to generate the classification model. The detail of each model is presented in Supplementary Table S6. Here we used all of classification models, which are available from the Classification Learner app in the applications toolbox in MATLAB [19]. A training gene set was used to train all of models within the training set (N = 198) which were divided into four TNBC subtypes: BLIA (n = 54), BLIS (n = 60), MES (n = 47) and LAR (n = 37). In this study, we performed five-fold cross validation to evaluate the model performance. To identify the best model, the performance analysis of each model was measured in terms of accuracy, sensitivity (recall), specificity, Precision (positive predictive value) (PPV), negative predictive value (NPV), F1 score, and AUC [37]. These performance indicators were defined and computed as follows:

Accuracy = (TP + TN)/(TP + FP + FN + TN). Sensitivity (Recall) = TP/(TP + FN) Specificity = TN/(TN + FP) Precision (positive predictive value (PPV)) = TP/(TP + FP) Negative predictive value (NPV)) = TN/(TN + FN) F1 score = 2(Precision* Recall)/(Precision + Recall) where, TP = true positive, TN = true negative, FN = false negative, FP = false positive.

Finally, the classification model which gave the best performance indicators for each TNBC subtype was selected for model generation and evaluation. 

### 4.5. Modeling Prediction and Performance Evaluation

After model training, 334 unknown TNBC cases were used to evaluate the prediction ability of the best performing model. There is no standard method to classify TNBC, so after making the predictions we identified the upregulated DEGs using mavolcanoplot in MATLAB for each TNBC subtype, to compare with the training set.

## 5. Conclusions

We proposed a new ML model to distinguish the four subtypes of TNBC using subtype-specific gene signatures based on gene expression data. Our finding confirmed that the SVM model offered the best potential classifier for TNBC classification. The utilization of a training gene set could be beneficial for TNBC subtyping and the development of personalized treatment for TNBC patients.

## Figures and Tables

**Table 1:** Top 20 unique upregulated DEGs of each TNBC subtype.

BLIA		BLIS		MES		LAR	
DEGs	FC	DEGs	FC	DEGs	FC	DEGs	FC
IDO1	4.41	GABRP	6.97	ADIPOQ	16.63	PIP	14.29
CXCL13	4.13	SOX8	5.49	ADH1B	14.32	FOXA1	12.52
MMP12	3.69	SHC4	4.51	OGN	10.44	DHRS2	10.59
LAMP3	3.60	ROPN1	4.21	CD36	9.46	TFF1	10.27
CXCL11	3.49	PROM1	4.17	FABP4	9.40	MLPH	9.97
GZMB	3.43	KRT14	3.89	CHRDL1	9.21	AGR3	9.70
RARRES1	3.40	KRT81	3.86	CFD	8.50	TFF3	9.48
CXCL10	3.40	HORMAD1	3.75	LINC01279	8.05	NAT1	8.27
CHI3L1	2.85	VTCN1	3.74	PLIN1	7.49	AGR2	8.01
AIM2	2.81	SOSTDC1	3.57	SFRP4	7.15	C9orf152	7.87
NUF2	2.71	PNMA8A	3.50	ACKR1	7.11	SCUBE2	6.99
TTK	2.70	IRX1	3.47	IGF1	5.95	GATA3	6.86
CXCL9	2.66	UGT8	3.38	HBB	5.86	SIDT1	6.79
APOBEC3B	2.65	KRT23	3.31	EFEMP1	5.84	REEP6	6.60
MCM10	2.63	ART3	3.24	GPX3	5.82	MUCL1	6.37
GBP5	2.62	ELF5	3.23	CXCL14	5.51	AR	6.20
EZH2	2.61	MIA	3.21	ENPP2	5.32	TOX3	5.90
CCL5	2.60	TTYH1	3.15	SRPX	5.27	GPR160	5.32
ADAMDEC1	2.55	PTPRZ1	3.15	DPT	5.23	PRR15	5.25
CEP55	2.54	COL9A3	3.08	IL33	5.19	FAM110C	5.19

**Table 2:** Comparison of classification accuracy of all algorithms using 719 training genes.

Classification Methods		% Accuracy			
		BLIA	BLIS	MES	LAR
SVM	Linear SVM	92.3 ± 0.4	93.6 ± 0.5	94.8 ± 0.2	98.0 ± 0.3
	Quadratic SVM	93.9 ± 0.7	93.4 ± 1.1	94.5 ± 0.2	98.4 ± 0.2
	Cubic SVM	94.2 ± 1.2	93.9 ± 1.0	94.4 ± 0.4	98.4 ± 0.2
	Fine Gaussian SVM	72.7 ± 0.0	69.7 ± 0.0	76.3 ± 0.0	81.3 ± 0.0
	Medium Gaussian SVM	95.7 ± 0.7	95.6 ± 0.7	95.0 ± 0.3	98.3 ± 0.2
	Coarse Gaussian SVM	74.2 ± 0.0	83.1 ± 0.4	91.4 ± 0.5	98.8 ± 0.2
KNN	Fine KNN	89.0 ± 1.0	88.5 ± 0.7	93.3 ± 0.4	98.4 ± 0.2
	Medium KNN	92.3 ± 0.8	93.4 ± 0.4	93.5 ± 0.4	97.8 ± 0.4
	Coarse KNN	72.7 ± 0.0	69.7 ± 0.0	76.3 ± 0.0	81.3 ± 0.0
	Cosine KNN	94.8 ± 0.8	94.3 ± 1.3	94.7 ± 0.6	98.5 ± 0.4
	Cubic KNN	92.1 ± 1.0	93.8 ± 0.7	93.9 ± 0.5	97.6 ± 0.8
	Weighted KNN	94.1 ± 0.8	93.7 ± 0.8	94.7 ± 0.5	98.1 ± 0.2
Ensemble	Boosted Trees	72.7 ± 0.0	69.7 ± 0.0	76.3 ± 0.0	81.3 ± 0.0
	Bagged trees	86.6 ± 1.8	88.5 ± 1.0	90.7 ± 1.4	92.9 ± 0.8
	Subspace Discriminate	91.1 ± 1.9	91.5 ± 1.1	92.2 ± 1.1	98.0 ± 0.5
	Subspace KNN	90.5 ± 1.0	90.3 ± 1.3	93.8 ± 0.2	98.4 ± 0.2
	RUSBoosted Trees	88.2 ± 1.8	88.9 ± 0.9	92.4 ± 0.7	95.4 ± 0.6
Tree	Fine Tree	82.3 ± 2.1	79.0 ± 2.5	89.7 ± 1.6	95.5 ± 1.6
	Medium Tree	81.7 ± 2.2	79.0 ± 2.5	89.7 ± 1.6	95.5 ± 1.6
	Coarse Tree	83.5 ± 1.7	80.9 ± 2.4	89.9 ± 1.8	95.5 ± 1.6
Linear Discriminant	Linear Discriminant	86.0 ± 1.2	90.2 ± 0.8	90.2 ± 1.1	97.4 ± 0.6
Logistic Regression	Logistic Regression	64.7 ± 1.7	61.5 ± 2.9	61.9 ± 4.0	74.0 ± 3.4
Naïve Bayes	Gaussian Naïve Bayes	76.8 ± 0.8	78.4 ± 0.2	95.0 ± 0.5	97.9 ± 0.2
	Kernel Naïve Bayes	83.0 ± 0.9	79.3 ± 0.4	95.0 ± 0.9	97.5 ± 0.6

**Table 3:** Overall performance of four selected classification models.

	BLIA	BLIS	MES	LAR
	Medium Gaussian SVM	Medium Gaussian SVM	Medium Gaussian SVM	Coarse Gaussian SVM
Recall	85.2	88.3	93.2	100.0
Specificity	99.3	97.1	96.1	98.8
Precision (PPV)	97.9	93.0	87.2	94.6
Negative Predictive value	94.7	95.0	98.0	100.0
F1 score	0.91	0.91	0.90	0.97

**Table 4:** Upregulated DEGs of unclassified samples.

Unclassified Samples	
Gene	FC
EN1	2.33
PROM1	2.11
CCL2	2.09

## Data Availability

Expression profile of GSE76124, GSE95700, GSE48390, GSE76275, GSE19697, GSE 20711 and GSE21653 in the manuscript was downloaded from the Gene Expression Omnibus (GEO) database (https://www.ncbi.nlm.nih.gov/geo/, accessed on 1 May 2021). We have provided our code implementation and trained models at https://github.com/Rassanee/TNBC, accessed on 1 May 2021.

## Supplementary Materials

The following are available online at https://www.mdpi.com/article/10.3390/jpm11090881/s1, Table S1: List of a training gene set; Table S2: Functional and pathway enrichment analysis of upregulated DEGs; Table S3: Model performance; Table S4: Prediction of test set; Table S5: Comparison of up-DEGs between training and test set; Table S6: The detail of the classification model.
