# Supplementary material for: Molecular Classification Models for Triple Negative Breast Cancer Subtype Using Machine Learning
Source: J Pers Med. 2021 Sep 1;11(9):881. doi: 10.3390/jpm11090881 (PMC8472680; doi:10.3390/jpm11090881)
Supplement: Supplementary file 1 [file jpm-11-00881-s001.zip › Supplementary Table S6.pdf]

Supplementary Table S6

Classification Method	Classifier Type	Parameters	Model Flexibility	Prediction Speed
SVM	Linear SVM	Linear kernel function	Low	Fast
	Quadratic SVM	Quadratic kernel function	Medium	Fast
	Cubic SVM	Cubic kernel function	Medium	Fast
	Fine Gaussian SVM	Gaussian kernel function with kernel scaling set to 1/4	Medium	Fast
	Medium Gaussian SVM	Gaussian kernel function with kernel scaling set to 1	Medium	Fast
	Coarse Gaussian SVM	Gaussian kernel function with kernel scaling set to 4	Medium	Fast
KNN	Fine KNN	The number of neighbors is set to 1.	Medium	Medium
	Medium KNN	The number of neighbors is set to 10.	Medium	Medium
	Coarse KNN	The number of neighbors is set to 100.	Medium	Medium
	Cosine KNN	Cosine distance metric with the number of neighbors set to 1.	Medium	Medium
	Cubic KNN	Cubic distance metric with the number of neighbors set to 10.	Slow	Medium
	Weighted KNN	Distance weight with the number of neighbors set to 10.	Medium	Medium
Ensemble	Boosted Trees	AdaBoost with decision tree learners of 10 maximum splits	Medium to High	Fast
	Bagged Trees	Bag with decision tree learners of 10 maximum splits	High	Medium
	Subspace Discriminant	Subspace with linear discriminant learners	Medium	Medium
	Subspace KNN	Subspace with nearest neighbor learners	Medium	Medium
	RUSBoost Trees	RUSBoost with decision tree learners of 10 maximum splits	Medium	Fast
Decision Trees	Coarse Tree	The maximum number of split is 4.	Low	Fast
	Medium Tree	The maximum number of split is 20.	Medium	Fast
	Fine Tree	The maximum number of split is 100.	High	Fast
Linear Discriminant	Linear Discriminant	The same covariance matrix for all classes and a gamma value of 1	Low	Fast
Logistic Regression	Logistic Regression	L2 Regularization	Low	Fast
Naïve Bayes	Gaussian Naïve Bayes	Data distribution set to Gaussian Distribution	Low	Medium
	Kernel Naïve Bayes	Data distribution set to kernel smoothing density estimate	Medium	Slow
